# Habitat, species richness and hantaviruses of sigmodontine rodents within the Interior Atlantic Forest, Paraguay

**DOI:** 10.1371/journal.pone.0201307

**Published:** 2018-08-01

**Authors:** Gillian Eastwood, Jeremy V. Camp, Yong Kyu Chu, Aubrey M. Sawyer, Robert D. Owen, Xueyuan Cao, Mariah K. Taylor, Leonardo Valdivieso-Torres, Richard D. Sage, Ashley Yu, Doug G. Goodin, Vicente J. Martinez Bruyn, Ryan C. McAllister, Laura Rodriguez, Evan P. William, Colleen B. Jonsson

**Affiliations:** 1 Department of Microbiology, University of Tennessee-Knoxville, Knoxville, TN, United States of America; 2 Department of Microbiology and Immunology, University of Louisville, Louisville, KY, United States of America; 3 Center for Predictive Medicine for Biodefense and Emerging Infectious Diseases, University of Louisville, Louisville, KY, United States of America; 4 Centro para el Desarrollo de la Investigación Científica, Asunción, Paraguay; 5 Department of Biological Sciences, Texas Tech University, Lubbock, TX, United States of America; 6 Department of Nursing-Acute/Tertiary Care, University of Tennessee Health Science Center, Memphis, Tennessee, United States of America; 7 Department of Microbiology, Immunology and Biochemistry, University of Tennessee Health Science Center, Memphis, Tennessee, United States of America; 8 Sociedad Naturalista Andino Patagónica (SNAP), Bariloche, Río Negro, Argentina; 9 Department of Geography, Kansas State University, Manhattan, KS, United States of America; 10 Facultad de Ciencias Exactas y Naturales, Universidad Nacional de Asunción, San Lorenzo, Paraguay; 11 Department of Pharmacology and Toxicology, University of Louisville, Louisville, KY, United States of America; 12 Fundación Moisés Bertoni, Asunción, Paraguay; Wistar Institute, UNITED STATES

## Abstract

Four of the nine sigmodontine tribes have species that serve as reservoirs of rodent-borne hantaviruses (RBO-HV), few have been studied in any depth. Several viruses have been associated with human cases of hantavirus pulmonary syndrome often through peridomestic exposure. Jabora (JABV) and Juquitiba (JUQV), harbored by *Akodon montensis* and *Oligoryzomys nigripes*, respectively, are endemic and sympatric in the Reserva Natural de Bosque Mbaracayú (RNBM), Paraguay, a protected area of the Interior Atlantic Forest. Rodent communities were surveyed along a 30 km stretch of the RNBM in eight vegetation classifications (Low, High, Bamboo, Riparian and Liana Forests, Bamboo Understory, Cerrado, and Meadow/Grasslands). We collected 417 rodents from which 11 species were identified; *Akodon montensis* was the predominant species (72%; 95%CI: 64.7%-76.3%), followed by *Hylaeamys megacephalus* (15% (11.2%-18.2%)) and *Oligoryzomys nigripes* (9% (6.6%-12.4%)). We examined the statistical associations among habitat (vegetation class) type, rodent species diversity, population structure (age, sex, and weight), and prevalence of RBO-HV antibody and/or viral RNA (Ab/RNA) or characteristic *Leishmania* tail lesions. Ab/RNA positive rodents were not observed in Cerrado and Low Forest. *A*. *montensis* had an overall Ab/RNA prevalence of 7.7% (4.9%-11.3%) and *O*. *nigripes* had an overall prevalence of 8.6% (1.8%-23.1%). For *A*. *montensis*, the odds of being Ab/RNA positive in High Forest was 3.73 times of the other habitats combined. There was no significant difference among age classes in the proportion of Ab/RNA positive rodents overall (p = 0.66), however, all 11 RNA-positive individuals were adult. Sex and habitat had independent prognostic value for hantaviral Ab/RNA in the study population; age, presence of tail scar/lesion (19% of the rodents) and weight did not. Adjusting for habitat, female rodents had less risk of becoming infected. Importantly, these data suggest habitat preferences of two sympatric rodent reservoirs for two endemic hantaviruses and the importance of including habitat in models of species diversity and habitat fragmentation.

## Introduction

Hantaviruses (genus *Orthohantavirus*, family *Hantaviridae*, order *Bunyavirales*) are negative-sense, single-stranded, tripartite RNA viruses found in mice, rats, voles, shrews, moles, and bats [1]. While there is no overt pathology in studies of natural reservoirs of hantaviruses, a negative impact of infection has been suggested in terms of weight gain, movement and survival of *Peromyscus maniculatus* infected with Sin Nombre virus (SNV) [2–5]. At least ten species of rodent-borne hantaviruses (RBO-HV) are associated with serious illness and death in human populations, globally [6, 7]. Not all RBO-HV have been associated with human disease. In South America, hantaviruses harbored by the Sigmodontinae have been associated with hantavirus pulmonary syndrome (HPS) [6]. RBO-HV have been discovered in four of the 9 tribes within the Sigmodontinae (Sigmodontini, Oryzomyini, Akodontini and Phyllotini), which include the majority of the approximately 370 Sigmodontine species identified in South America [8, 9]. In the Southern Cone, HPS cases have been attributed to Andes hantavirus (ANDV, reservoir *Oligoryzomys longicaudatus*) in Chile and Argentina. Laguna Negra hantavirus (LANV, reservoir *Calomys laucha*) has been identified as the cause of HPS in western Paraguay, Bolivia and southern Brazil. In eastern Paraguay, Uruguay and Brazil, within the Interior Atlantic Forest (IAF), two closely related strains of hantaviruses, Juquitiba (JUQV) and Araucaria (ARAUV), associated with *Oligoryzomys nigripes*, have been linked to HPS cases in Brazil and Argentina [10, 11]. Notably, the mortality associated with ANDV/JUQV and LANV differ with LANV cases showing a lower case fatality (15 versus 40% for ANDV) [12–15]. To our knowledge, no cases of HPS have been associated with Jabora virus (JABV), harbored by *Akodon montensis* (tribe Akodontini). *Akodon montensis* is widely distributed in the IAF, a vast neotropical biome that extends into eastern Paraguay, and into the temperate grass and shrubland biomes of Argentina [16, 17]. However, another member of the Akodontini (*Necromys lasiurus*) has been associated with HPS cases associated with Araraquara virus in Brazil [18].

The diversity of the South American RBO-HV mirrors that of the Sigmodontinae with numerous lineages across a wide range of ecologically diverse habitats. For example, the closely related, LAN, Rio Mamoré, and Alto Paraguay hantaviruses are each carried by different rodent genera that inhabit the tropical grass and shrublands, and dry broadleaf forest areas along a large expanse of the western and central regions of South America in Peru, Bolivia, Brazil and Paraguay [13, 19–22]. In the moist broadleaf forest biomes from Venezuela and Colombia into Paraguay, distinct rodent genera harbor JABV, Maporal or Necocli hantaviruses [12, 23–25]. In the IAF and coastal Atlantic Forest in Brazil, several phylogenetically distinct lineages of RBO-HV, (e.g., JUQV, Oran and Lechiguanas hantaviruses), have been identified in a variety of *Oligoryzomys* species [16, 17]. In the Reserva Natural del Bosque Mbaracayú (RNBM) in eastern Paraguay, the reservoirs of JABV and JUQV, *Akodon montensis* and *Oligoryzomys nigripes* respectively, are sympatric [12]. Other reservoirs of RBO-HV have been observed in the RNBM such as *Oligoryzomys mattogrossae* and *Necromys lasiurus*; however, the strains reported in these species have not yet been detected [26, 27]. The diversity of rodents and RBO-HV in South America makes this a compelling biogeographic region to study the ecology and evolution of RBO-HV and their reservoir rodent hosts.

JABV has been reported in *A*. *montensis* in Paraguay, and neighboring Brazil, with an overall hantaviral antibody prevalence of 10% and 14.5%, respectively [12, 23, 28]. JUQV is present in *O*. *nigripes* within Paraguay, Argentina, Uruguay, and Brazil, with a reported antibody prevalence ranging from 3–12% [12, 29]. These rodent species are broadly sympatric, yet have distinct ecological niches; for example, *Oligoryzomys* species are arboreal and ground dwelling whereas *Akodon* species are ground dwelling. We and others have observed both spatial and temporal sympatry among hantavirus species-strains and their reservoirs, suggesting that intergeneric spillover may be possible [30, 31]. Spillover infections of these hantaviruses in related mouse species have been detected in Brazil; with JABV reported in *Akodon paranaensis* and *Akodon serrensis*, and JUQV reported in *A*. *paranaensis* and *Oxymycterus* species [32, 33]. Previously, we reported spillover of JUQV in *O*. *mattogrossae* (originally reported as *O*. *fornesi*) [13, 14, 31]. In addition, ARAUV, has been detected by RT-PCR in *A*. *montensis*. *A*. *paranaensis*, *O*. *nigripes*, and *Oxymycterus judex* [34]. Spillover infection in secondary hosts may play an important role in maintaining hantaviruses such as JABV and JUQV in nature given their low prevalence (and R_0_) in wild reservoir populations [30–32].

We conducted a survey of rodent communities at 22 sites across a 30 km cross-section of the RNBM in eastern Paraguay. The RNBM contains the largest, protected remnant of the IAF of late-stage second-growth forest with minimal human impact during the last 35 years. The only approved uses of the RNBM are hunting (by traditional methods) by the indigenous Ache people, a small amount of biological research, and ecotourism. We collected rodents in eight vegetation classes present in the RNBM; Low, High, Bamboo and Liana Forests, Bamboo Understory, Cerrado, Riparian and Meadow/Grasslands (**Table 1**). These classifications are based on vegetation classifications reported by Naidoo and Hill [35]. We report on the distribution of these rodents within these habitat types and using a logistic regression framework report on the association of sex, age, weight, habitats and characteristic tail lesion/scars with the prevalence of hantaviral Ab/RNA. Sex and habitat had independent prognostic value for hantaviral Ab/RNA in the study population with female rodents showing significant lower risk of becoming infected compared to male rodents. Of the eight habitats, the odds of *A*. *montensis* being infected in High Forest was 3.73 times of the other habitats combined. These results underscore the importance of habitat and sex in the study of hantaviral prevalence, maintenance and ecology.

**Table 1:** Vegetation classifications within the RNBM.

Class(Habitat)^a^	Description	Proportion represented at RNBM
Bamboo Forest	Bamboo forms the main vegetation structure and canopy	8.2%
Bamboo Understory	Interior understory canopy filled with dense patches of *Chusquea* species bamboo	22.9%
Cerrado/Cerradon	Mixed, predominantly savannah, but also pure pasture, grassland with palms or woody shrubs.	5.1%
High Forest	Primary tall forest, tree height ranges around 20m, with open sub-canopy	30.2%
Low Forest	Tree height less than 10-15m, Myrtaceae species, often near water courses.	7.0%
Liana Forest	Forest with abundant lianas predominating in the understory	13.8%
Meadow/Grassland	Open area dominated with native grasses	2.4%
Riparian	Woodlands and vegetation associated with rivers or streams	9.1%

^**a**^An additional 1.3% of the area is under restoration.

## Methods

### Rodent collection

**Fig 1:**
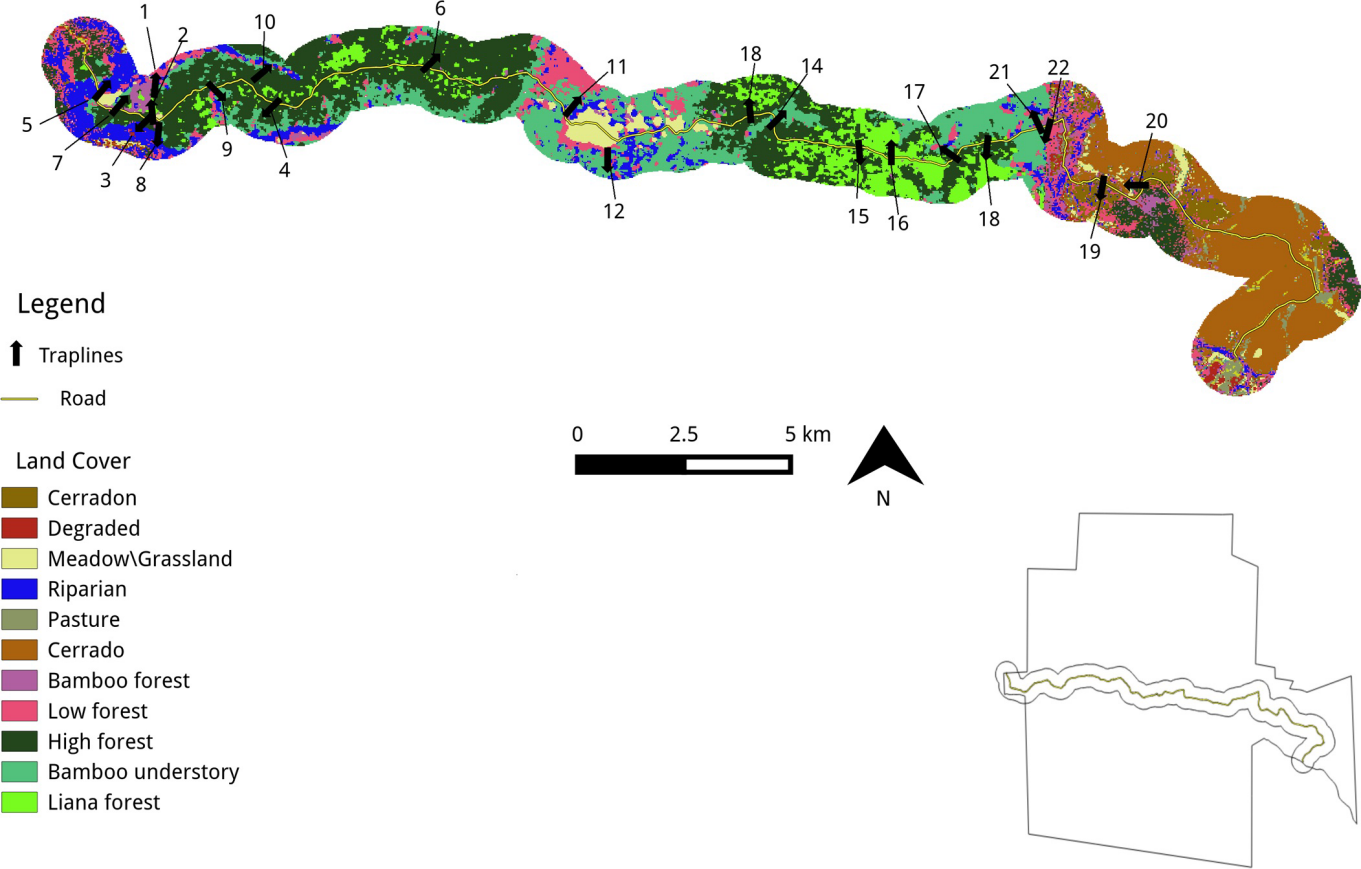
Study region of RNBM highlighting locations of transects and habitat type. Land cover types shown for a corridor through RNBM (inset shows an outline of the park and transect location) in which the trapping sites were located. Vegetation classes were derived from Naidoo and Hill, 2006.

ArcGIS [36] was used to identify 22 perpendicular transects (**Fig 1**) from a dirt road extending ca. 30 km from west-to-east for capture studies of rodents (**Table 1**). The transects were selected with the objective of sampling as many vegetation classes (habitat types) as possible across the RNBM. Most of the transects required clearing with a machete. This was done on the first day as the traps were placed and set.

During August 2014, fifty Sherman traps (7.6 x 8.9 x 22.9 cm, Sherman Trap Company, Tallahassee, FL) were set, 10 m apart, along each of the 500-m transects. Between 20–30% of the traps were placed 1–3 m above ground, on branches or in vines as the habitat permitted, and the remainder were on the ground. Because our primary objective was to collect as many mice from as many habitats and regions of the forest reserve as possible, we had nine lines of 50 traps each open on most nights. These were staggered across the 22 collecting sites as effectively as possible (See **S1 Table** for GIS coordinates of lines and trapping approach). For logistical reasons, fewer lines were open during the first three nights and last night of the collecting period. We ran each line for 2–11 nights, for a total of 167 line-nights, or 8,350 trap-nights. Ten additional smaller lines (generally with 10 traps per line) were also set to sample less extensive habitat types (e.g., Riparian). These traps were generally kept open for three nights, so these lines contributed an additional ca. 300 trap-nights to our sampling effort. Each capture site with one or more captures was categorized as one of the eight habitat types (**Table 1**).

Each rodent was identified morphologically to species, and weight, total length, tail, ear, and hind foot lengths were measured. Specimens were identified following D’Elía and Pardiñas (2015) and authors therein [8]. The sex, reproductive status and categorical age (juvenile, sub-adult, adult) were also recorded. Liver, lung, heart, kidney, muscle, spleen, colon, blood, urine, saliva and embryos (when encountered) were harvested and stored immediately in liquid nitrogen, prior to transport to the USA, where the samples were stored at -140°C until processing. Any visible clinical signs, such as enlarged spleen, liver parasites, or tail scars (prominent at the top of the base of the tail) were recorded during necropsy. Lesions and/or scarring, potentially from *Leishmania* infection, occurred primarily on the tail, but also occasionally on ears or feet. These lesions are visually characteristic (see for example [37, 38], and could not easily be confused with wounds from other sources (e.g., injury, bite). Wounds from other sources were not included. All voucher specimens will be deposited in the Museo Nacional de Historia Natural del Paraguay, or another accredited Paraguayan research collection. At present all voucher specimens are held in an authorized collection (Paraguayan Secretaria del Ambiente, Habilitación No. 004/2015) of Dr. Robert Owen. Tissue samples, not consumed from this research and other on-going projects, will be deposited with the Natural Science Research Laboratory at the Museum of Texas Tech University.

### Molecular confirmation of rodent species

Cytochrome c oxidase subunit I (COI) or cytochrome b (Cytb) gene sequence was used to confirm the identification of the *Oligoryzomys* and selected *Akodon* and *Hylaeamys* specimens. Total genomic DNA was isolated from rodent muscle, kidney or spleen using DNAzol and homogenization with the Omni Tissue Bead Mill 4 (Invitrogen). A 639-bp fragment of the COI, and/or a 1140-bp fragment of the Cytb gene, was amplified via PCR in 50 μL reactions containing 1X Phusion High-Fidelity PCR Master Mix, 0.5 μM of each forward and reverse primer, and 50 ng of DNA. For the COI gene, we used COF1 (5’-CTTATCTTYGGTGCYTGAG-3’) and COR1 (5’-CCAAAAAATCAGAATAGGTGTTG-3’). The complete CytB gene was amplified using primers Mus14095 (5'-GACATGAAAAATCATCGTTGTAATTC) [39] and Mus587-612R (5'-GTCTGAGTTTAAGCCTGATGGG), and sequenced with these primers and Mus418-435 (5'-CCCATCAGGMTTAAACTC) and Mus471-494R (5'—GAATGCCYARTARATCYTTRATTC’). Thermocycler conditions for COI primers were: initial denaturation at 98°C for 30 s, followed by 35 cycles of 98°C for 10 s, 58°C for 20 s, and 72°C for 30 s, followed by a final extension at 72°C for 5 min. Conditions for Cytb primers were: denatured at 95°C for 45 s, primers were annealed at 50°C for 40 s, and segments were extended at 70°C for 2 min with 4 s added to each extension for 40 cycles with a final extension at 70°C for 5 min. PCR-products were visualized using E-gels (Invitrogen), purified using the Wizard SV Gel and PCR Clean-Up System (Promega), and sequenced via University of Tennessee Genomics Core or Eurofins Genomics using the Sanger method. Sequences were aligned using MUSCLE, and visualized/trimmed in MEGA7 [40]. The identity of each specimen was assessed using cleaned sequences by BLAST and phylogenetic methods using maximum likelihood inferred in MEGA7 or RAxML-HPC BlackBox on the CIPRES Science Gateway V. 3.3 [41] with GTR + I + G as the evolutionary model [42], 1000 bootstraps. Node values greater than 70% were considered statistically robust.

### Immunofluorescence assay (IFA)

An indirect IFA was used to screen blood samples for antibodies that cross-reacted with ANDV grown in Vero E6 cells as described previously [14]. Briefly, blood samples were diluted 1:10 in PBS and incubated at room temperature on acetone-fixed, 10-spot slides with antigen. The secondary antibody, a 1:1 mixture of FITC-labeled anti-rat IgG and FITC anti-mouse IgG (both Kirkegaard and Perry Laboratories, Inc.), was applied to identify antibody-positive wells. Positive blood samples were titrated to end-point by a two-fold serial dilution of blood from 1:32 through 1:4096 in duplicate and tested as above.

### Screening of rodent lung RNA by reverse-transcriptase polymerase chain reaction (RT-PCR) for JABV and JUQV and Sanger sequencing

Total RNA was isolated from all available rodent lung specimens following homogenization in TRIzol Reagent (Invitrogen) with an Omni Tissue Homogenizer (Fisher Scientific) as described previously [13]. cDNA was synthesized using SuperScript® III First-Strand Synthesis System (Thermo Scientific) using JABV and JUQV S-segment primers, and PCR-amplified using Phusion High-Fidelity PCR Master Mix (Thermo Scientific) with HF buffer [14]. Of the eleven RT-PCR samples, quality sequence was obtained from five using the Sanger method as described above.

### NexGen sequencing and phylogenetics

For NexGen sequencing, cDNAs were made for TK121843, TK121861, TK184520, TK184640, and TK184699, using a mixture of ANDV, JABV, and JUQV S-segment primers (sequences available upon request) with the SuperScript IV Reverse Transcriptase Kit (Thermo Fisher). S-segment was amplified with Phusion High-Fidelity PCR Master Mix with HF Buffer (Thermo Fisher) and an equimolar mixture of ANDV, JABV, and JUQV primers mixture (available upon request) at 95°C for 2 min and 30 cycles at 95°C-30s, 60°C-30s and 72°C for 1 min. Sequencing libraries were created using Nextera XT Library Prep kit according to the Illumina protocol except that tagmentation time was extended to 10 min, which gave a better tagmentation product. Libraries were assessed for quality using High Sensitivity DNA Chips (Agilent) on an Agilent 2100 Bioanalyzer. Quantification was further verified using KAPA Biosystems Quantitation of next generation sequencing library preparation kit, following the manufacturer’s protocol. All libraries were pooled into a final concentration of 0.5 nM and loaded onto a MiSeq using the MiSeq Reagent Kit v3 600 cycles. CLC Genomics Workbench v8.5 (Qiagen) was used to analyze the sequencing reads. Reads were filtered by exclusion of broken pairs and aligned to S, M, and L segment reference genomes of ANDV AY267347.1, AY363179.1, and EU788002.1, respectively, and the S segment reference genomes of JABV GU205329.1, and JUQV GU213198.1 using the Map Reads to Reference tool. The consensus sequence of TK121861 and TK184699 which had identical amino acid sequence similarities to JABV and JUQV, respectively, were extracted using a threshold of 10 and regions of low coverage were removed. All sequences were deposited at GenBank (MG575411-5). We aligned 292 nt of each sequence using MUSCLE and examined relationships by maximum likelihood using MEGA 7.

### Statistical analyses

Descriptive statistics were used to describe the distribution of captured rodents and prevalence of hantavirus antibody or RNA in rodents. Proportions with confidence intervals were calculated by binomial test. Chi-squared tests were used to address the association of rodent species with habitat types or hantavirus Ab/RNA detected and p values were derived from 10,000 Monte Carlo simulations. Shannon’s diversity index (H = -Σi = 1 pi ln pi) was calculated by vegan package in R and Shannon’s equitability was calculated as H/ln(S), where S is the species richness in that habitat or line. Univariate logistic regression was used to model the prevalence of hantavirus detected by hantaviral Ab/RNA with sex, age, weight, habitat, and characteristic tail scars/lesions as an independent variable. Akaike Information Criteria (AIC) was used to select a best fit multivariable model among all subset of the 5 predictors. Likelihood ratio test was used to assess the effect of a factor by comparing the logistic regression model with both factors and by comparing the logistic regression model with the model in which the factor was left out. All analyses were performed in R-3.4.3 (https://www.r-project.org/).

### Ethics statements

All animal procedures were approved (Approval No. 14024–03) by the Texas Tech University Institutional Animal Care and Use Committee (IACUC), which follows the 8th Edition of the Guide for the Care and Use of Laboratory Animals (Guide), NRC 2011, and the Animal Care and Use Committee guidelines of the American Society of Mammalogists for the use of wild mammals in research and education. The study did not involve endangered or protected species. Specimens were collected with approval of the Secretaría del Ambiente, Paraguay (Permiso de Caza o Colecta Científca No. 011/2014).

## Results

### Distribution of sigmodontine species by habitat

We captured 417 rodents representing nine genera and eleven species across the eight habitat types (**Tables 2 and S2**). Of the 417 mice, two mice had no IFA assessment and were excluded from subsequent analyses (**S1 Fig**). Three habitats (**Table 2**) had less than 10 captured mice (i.e. Cerrado, Liana Forest and Meadow/Grasslands) and were excluded in the association analyses involving habitat.

**Table 2:** Distribution of captured rodent species by habitat type.

	HABITAT TYPE*								
SPECIES	BF	BU	CE	HF	LF	LiF	MG	RV	Total
***Akodon montensis***	12	78	0	107	5	63	5	29	**299**
***Calomys callosus***	0	0	1	1	1	0	0	1	**4**
***Calomys tener***	1	0	1	0	0	0	0	0	**2**
***Cerradomys maracajuensis***	0	0	1	0	0	0	0	0	**1**
***Delomys dorsalis***	0	1	0	0	0	0	0	0	**1**
***Hylaeamys megacephalus***	0	22	0	16	2	12	3	5	**60**
***Nectomys squamipes***	0	0	0	0	0	0	0	2	**2**
***Oligoryzomys mattogrossae***	0	0	1	0	0	0	0	1	**2**
***Oligoryzomys nigripes***	3	13	0	12	0	2	0	8	**38**
***Scapteromys aquaticus***	0	1	0	0	0	0	0	0	**1**
***Sooretamys angouya***	0	5	0	0	0	0	0	0	**5**
**Total**	**16**	**120**	**4**	**136**	**8**	**77**	**8**	**46**	**415**

* Low Forest (LF), High Forest (HF), Bamboo Forest (BF) and Liana Forest (LiF), Bamboo Understory (BU), Cerrado (CE), Riparian Forest/Vegetation (RV) and Meadow/Grasslands (MG)

*Akodon montensis* was the predominant species (72%; 95%CI: 64.7%-76.3%) throughout the RNBM, followed by *Hylaeamys megacephalus* (15% (11.2%-18.2%)) and *Oligoryzomys nigripes* (9% (6.6%-12.4%)). The remaining species were captured much less frequently (**Table 2**). There was significant association between rodent species and habitats (*X*^2^ = 82.65, df = 36, p < 0.001, Cerrado, Liana Forest and Meadow/Grasslands were excluded).

### Evidence of hantaviral infection

Evidence of infection was suggested by the presence of antibodies and/or viral S-segment in 26 individuals (23 *A*. *montensis* and three *O*. *nigripes*). Based on the available samples for antibody and RNA measurements, the prevalence of antibody-positive *A*. *montensis* was 6.7% (4.1%-10.1%) and the prevalence of RNA-positive *A*. *montensis* (22 not tested, n = 277) was 2.9% (1.3%-5.6%); for an overall prevalence of 7.7% (4.9%-11.3%) (**Table 3**). Based on the available samples for antibody or RNA measurements, the prevalence of antibody-positive *O*. *nigripes* was 5.6% (0.7%-18.7%)) and the prevalence of RNA positive *O*. *nigripes* (four not tested, n = 34) was 8.8% (1.9%-23.7%); for an overall prevalence of 8.6% (1.8%-23.1%). In contrast to prior studies, no spillover was observed into *O*. *mattogrossae* (but only two individuals were captured). In summary, 26 of the 415 rodents (6.3% (4.1%-9%)) had antibody and/or RNA (**Table 3**). There was no significant evidence of association between rodent species with infection (p>0.05). Reciprocal IFA titers in whole blood ranged from 32 to 4096 with most rodents showing titers greater than 128 (**Table 4**).

**Table 3:** Presence of hantaviral antibody (Ab) in blood and/or viral RNA in lung blood or lung by rodent species.

SPECIES	Ab(+) RNA(+)	Ab(+) RNA(-)	Ab(-) RNA(+)	Negative	TOT
*Akodon montensis*	5	15	3	276	299
*Calomys callosus*	0	0	0	4	4
*Calomys tener*	0	0	0	2	2
*Cerradomys maracajuensis*	0	0	0	1	1
*Delomys dorsalis*	0	0	0	1	1
*Hylaeamys megacephalus*	0	0	0	60	60
*Nectomys squamipes*	0	0	0	2	2
*Oligoryzomys mattogrossae*	0	0	0	2	2
*Oligoryzomys nigripes*	2	0	1	35	38
*Scapteromys aquaticus*	0	0	0	1	1
*Sooretamys angouya*	0	0	0	5	5
Total	7	15	4	389	415

**Table 4:** Distribution of IFA Reciprocal titers in rodent reservoir species of JABV and JUQV.

	RECIPROCAL TITERS				
SPECIES	32–64	128–256	512–1024	2048–5096	Total
*Akodon montensis*	1	2	8	9	20
*Oligoryzomys nigripes*	0	2	0	0	2
**Total**	**1**	**4**	**8**	**9**	22

**Fig 2:**
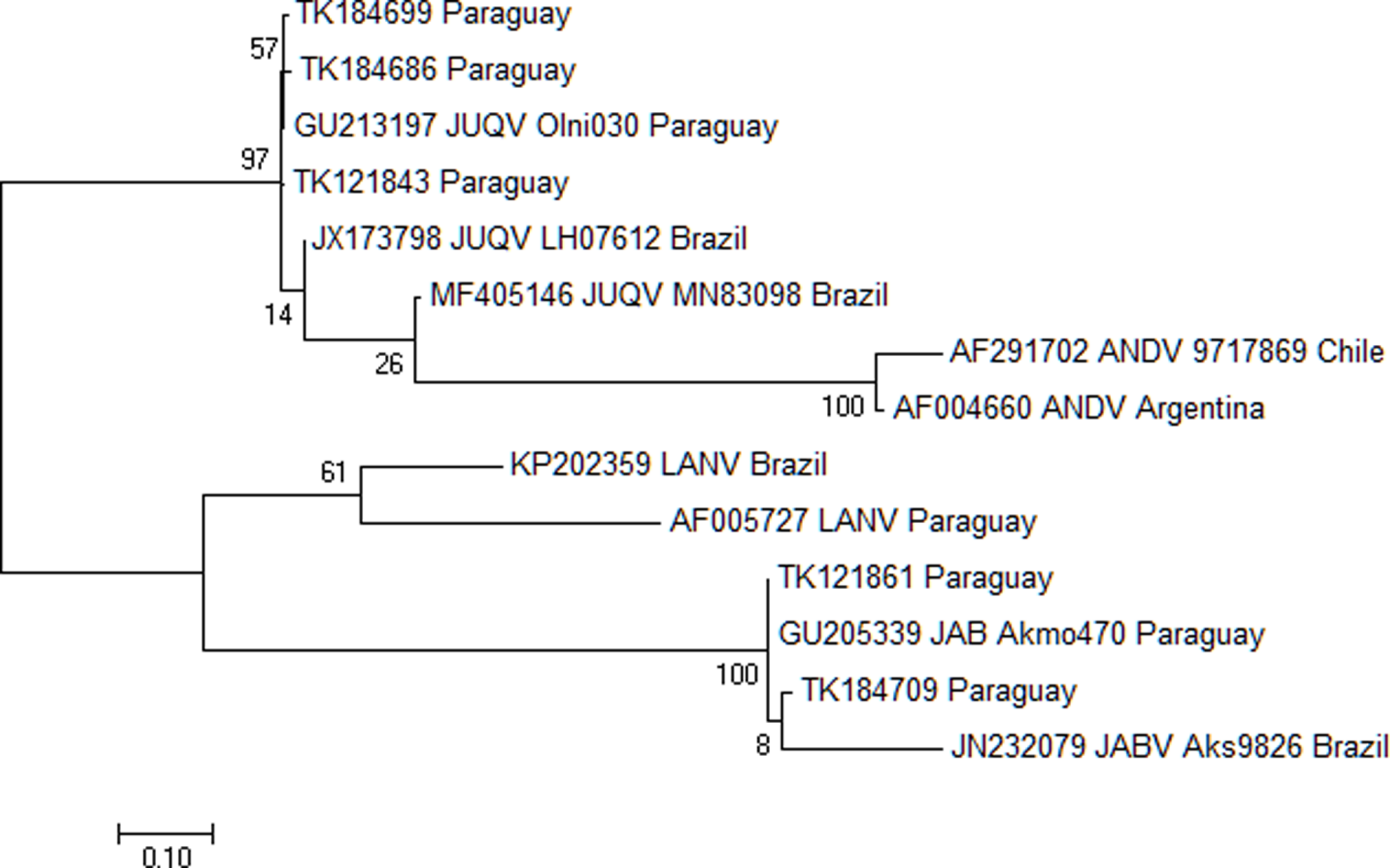
Molecular phylogenetic analysis of S segment of sequences from *A*. *montensis* and *O*. *nigripes*. Sequences from rodent lung specimens TK121843 TK184699, TK121861, TK184686 and TK184709 and GenBank references were analyzed using the Maximum Likelihood method based on the General Time Reversible model [43]. Reference sequences are shown with their GenBank reference number, virus and country from which they were isolated. The tree with the highest log likelihood (-1374.9790) is shown. The percentage of trees in which the associated taxa clustered together is shown next to the branches. Initial tree(s) for the heuristic search were obtained automatically by applying Neighbor-Join and BioNJ algorithms to a matrix of pairwise distances estimated using the Maximum Composite Likelihood (MCL) approach, and then selecting the topology with superior log likelihood value. A discrete Gamma distribution was used to model evolutionary rate differences among sites (5 categories (+*G*, parameter = 0.2861)). The rate variation model allowed for some sites to be evolutionarily invariable ([+*I*], 0.0010% sites). The tree is drawn to scale, with branch lengths measured in the number of substitutions per site. The analysis involved 14 nucleotide sequences. Codon positions included were 1st+2nd+3rd. There was a total of 251 positions in the final dataset. Evolutionary analyses were conducted in MEGA7.

Four of the RNA-positive rodents (three *A*. *montensis* and one *O*. *nigripes*) were negative for antibodies suggesting a recent infection. Of the 11 RNA-positive samples, S-segment sequences were obtained from five specimens with sequence identity to JUQV or JABV S-segments, based on phylogenetic analysis (**Fig 2**).

### Habitat association, species diversity and Ab/RNA prevalence of hantaviruses

We evaluated the prevalence of infection of hantavirus as determined by Ab/RNA positive rodent reservoirs within the habitats assigned for each trap (**Table 5**) and along each transect (**Table 6**). There was evidence of Ab/RNA in all types of habitat except Cerrado and Low Forest, both of which had only a few captures and while calculated for Shannon Index and Equitability, they were not considered significant due to small sample size (**Table 5**). The distribution of age and sex for the rodents found in each habitat are presented as well. Species diversity, calculated using the Shannon Equitability (considering habitats where sixteen or more rodents were captured), suggested that rodent diversity was highest in Meadow/Grassland EH = 0.954 > Riparian Vegetation (0.64) and lowest in High Forest (EH = 0.5) (**Table 5**). Comparing across habitats there was no apparent linear correlation between rodent species diversity and hantavirus-prevalence (percentage of positive animals) (Spearman correlation: -0.3; p = 0.68).

**Table 5:** Hantaviral Ab/RNA status in captured rodents by sex and age across habitat types.

HABITAT TYPE^a^ (% rodents)	Shannon Index H	Shannon Equit. E_H_	Juv Male	Juv Female	Juv Unk	Sub-adult Male	Sub-adult Female	Adult Male	Adult Female	Total
BF (3.9%)	0.703	0.64	0	0/1	0	0/1	0/1	1/5	0/8	1/16
BU (28.9%)	1.044	0.583	0/12	0/11	0	0/12	0/11	3/46	0/28	3/120
CE (1%)	1.386*	1*	0	0	0	0	0	0/2	0/2	0/4
HF (32.8%)	0.691	0.498	2/7	1/10	0	1/13	2/18	7/51	2/37	15/136
LF (1.9%)	0.9*	0.819*	0	0/1	0	0	0	0/2	0/5	0/8
LiF (18.6%)	0.549	0.499	0/9	0/6	1/3	0/4	0/12	2/24	1/19	4/77
MG (1.9%)	0.662*	0.954*	0	0	0	0/3	0	1/4	0/1	1/8
Riparian Veg (11.1%)	1.139	0.636	0/5	0/3	0	0/4	0/1	2/21	0/12	2/46
OVERALL	0.953	0.398	2/33 (6.1%)	1/32 (3.1%)	1/3 (33.3%)	1/37 (2.7%)	2/43 (4.7%)	16/155 (10.3%)	3/112 (2.7%)	26/415 (6.3%)

^**a**^The proportion of rodents in that habitat type rounded to the nearest whole number.

*- not significant due to small sample size

**Table 6:** Overall hantaviral (HV) Ab/RNA status and Shannon diversity in captured rodents by line.

TRANSECT LINE	06	07	09	10	13	14	15	17	Total
**HV-Negative**	41	14	45	17	21	43	60	22	389
**HV-Positive**	3	6	1	3		1	3		26
**Total No. Rodents**	44	20	46	20	21	44	63	22	415
**Proportion of HV-Positive**	0.07	0.30	0.02	0.15	0.00	0.02	0.05	0.00	
**SHANNON (H)**	0.493	0.999	0.790	0.518	0.864	0.878	0.517	1.032	

In rodent lung tissues and blood, Ab/RNA was detected in ten of the 22 transects sampled (lines 4, 6, 7, 9, 10, 11, 14, 15, 16, 21). Considering Shannon (H) diversity within transects with sufficient numbers of rodents (i.e., 6, 7, 9, 10, 13, 14, 15, 17) there was no apparent association between species diversity and the proportion of hantavirus-positive rodents (**Table 6**).

### Habitat association and prevalence of hantaviral Ab/RNA by rodent species

In examination of the Ab/RNA positive *A*. *montensis*, 15 were from High Forest (14% prevalence), four were from Liana Forest (6%), two were from Riparian Vegetation (7%), one from Bamboo Understory (1%), and one from Meadow-Grasslands (20%) (**Table 7**). The prevalence of JABV Ab/RNA in *A*. *montensis* was common in High Forest and Liana Forest types (11.2% (6.9%, 16.9%)) and was more frequent than the prevalence of JABV Ab/RNA in *A*. *montensis* in the other habitat types at the study site (3.1% (0.8%, 7.7%)). There was significant association between rodent habitats and infection in *A*. *montensis* (p = 0.047). For *A*. *montensis*, the odds of hantaviral Ab/RNA in High Forest was 3.73 times that of the other habitats combined (Odds ratio: 3.73; 95%CI: 1.42–10.55; p = 0.003).

**Table 7:** Association of Ab/RNA-positive *Akodon montensis* by habitat.

HABITAT TYPE *(%* of all *A*. *montensis)*	No.	% Ab/RNA Positive
Bamboo Forest	12 (4%)	0
Bamboo Understory	78 (26%)	1 (1%)
Cerrado/Cerradon	0	0
High Forest	107 (36%)	15 (14%)
Low Forest	5 (2%)	0
Liana Forest	63 (21%)	4 (6%)
Meadow/Grassland	5 (2%)	1 (20%)
Riparian Forest	29 (10%)	2 (7%)
*TOTAL*	*299*	23 (8%)

*Oligoryzomys nigripes* that were positive for Ab/RNA to JUQV were detected only in Bamboo Forest (1/3) and Bamboo Understory (2/13) (habitats that together represent 41% (16/38) of all capture sites; **Table 2**). *Oligoryzomys nigripes* was also captured in High (12/38), Liana (2/38) and Riparian (8/38) Forests. The significance of these associations was not determined given the small Ab/RNA abundance within each habitat.

### Association of population structure and prevalence of Ab/RNA in *A*. *montensis*

Because prior studies of hantaviral reservoir hosts have found age, weight and sex to be factors in infection prevalence (e.g., [44]), we examined the rodent population structure by weight, age and sex. The age and sex stratified population structure of the rodents showed that 53% of the 299 rodents were male, 47% were female and the sex of three juveniles was undetermined (**Table 8**). Evidence of hantavirus infection (Ab/RNA) was detected in a higher proportion of males (0.102) than females (0.043) (Odds Ratio: 2.51; 95%CI: 0.90–8.07; p = 0.074). Most of the rodents captured were adult (62%), with 58 sub-adults (19%), and 56 juveniles (19%). There was no significant difference among age classes in the proportion of Ab/RNA positive rodents overall (p = 0.66), however, all 11 RNA-positive individuals were adult.

**Table 8:** *Akodon montensis* sampled in RNBM by age, sex, and Ab/RNA prevalence.

	Total No. *A*.*m*.	Total No. Ab/RNA Pos *A*.*m*.	% Pos *A*.*m*.
**MALE**	** **	** **	
Juvenile	27	2	7
Sub-adult	25	1	4
Adult	105	13	12
**FEMALE**			
Juvenile	26	1	4
Sub-adult	33	2	6
Adult	80	3	4
**Sex** **unknown**			
Juvenile	3	1	0
**TOTAL**	** 299**	** 23**	**8**

**Fig 3:**
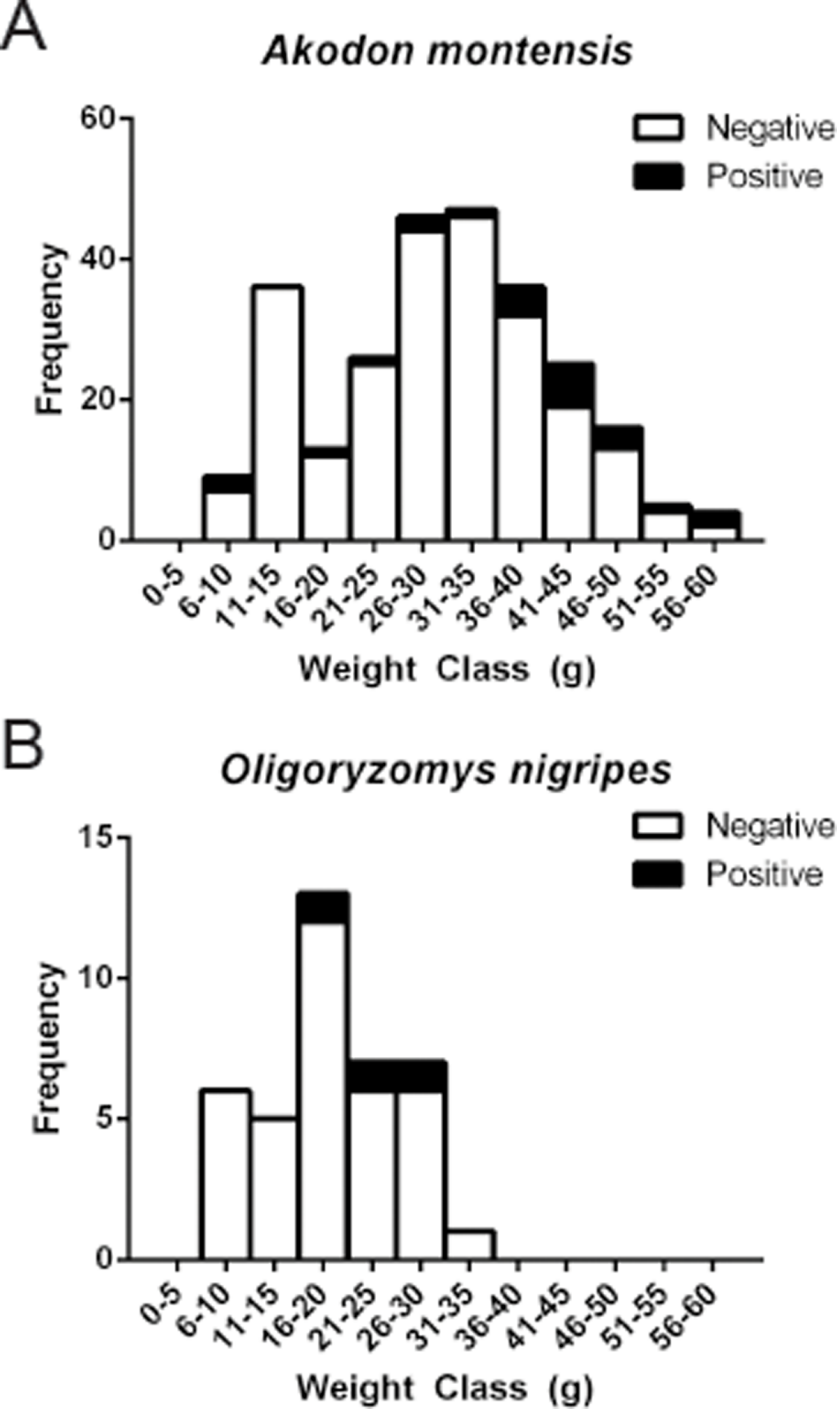
Weight range of adult rodents and percent hantavirus positive for *Akodon montensis and Oligoryzomys nigripes*. The number of mice that were negative for antibodies and/or viral RNA are shown in the bar graph by age grouping. The proportion of animals in each age group that were positive are shown in the darkened area of the bar.

The adult weight range of the two species in which Ab/RNA for JUQV and JABV was detected was 18-60g (*A*. *montensis*; n = 185) and 9-31g (*O*. *nigripes*; n = 33) (**Fig 3**). For *A*. *montensis*, weight was a significant factor in hantavirus prevalence (log odds of number of hantaviral positive rodents increased by 0.12 per g of weight, Z = 3.613, *P < 0*.*01*).

### Association of sex, age, weight, habitat, characteristic tail lesions with hantaviral Ab/RNA

**Fig 4:**
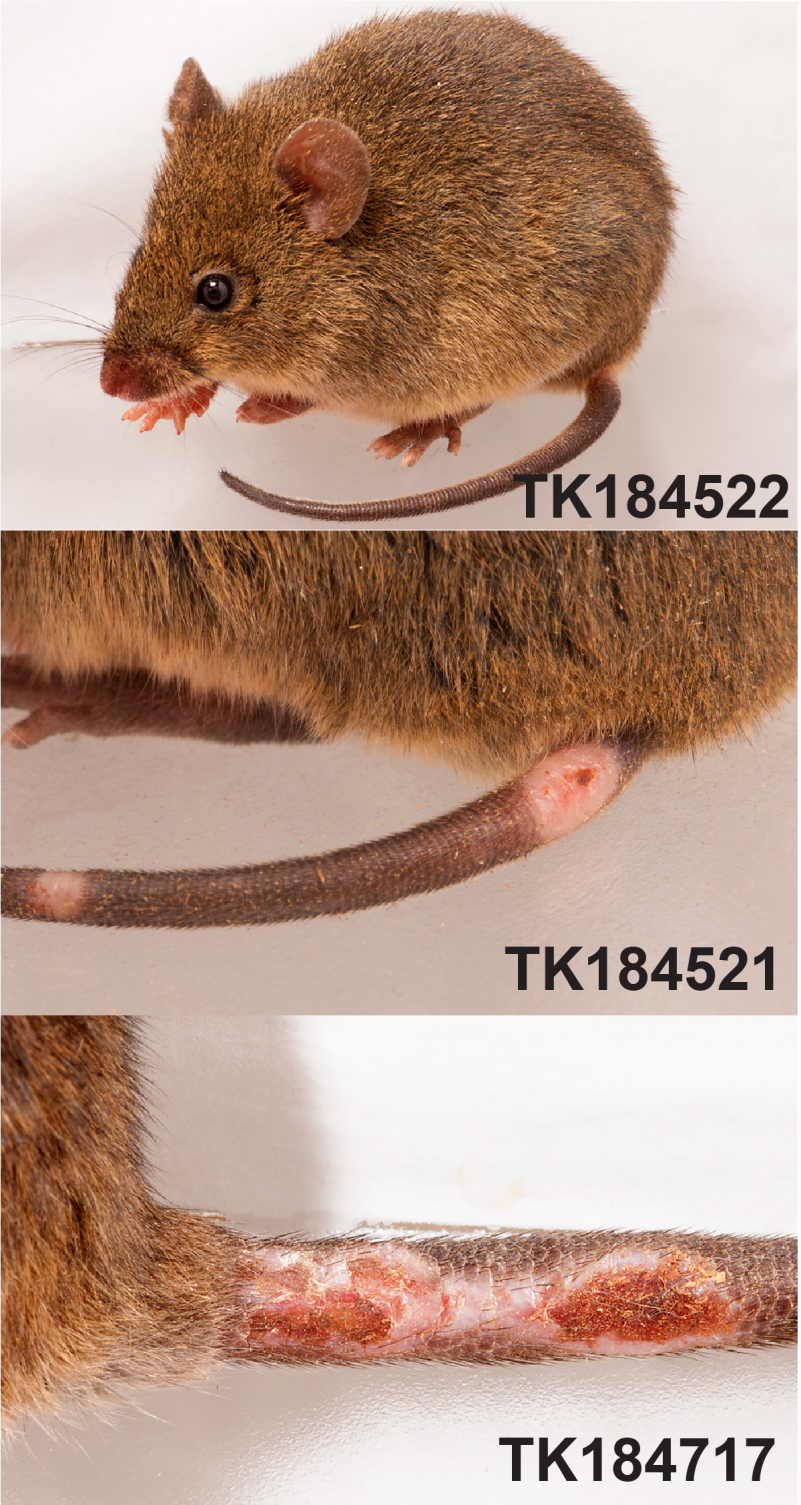
Representative photographs of lesions identified in rodents at the base of the tail. Photographs from adult, male mice are shown; TK184522 and TK 184221 are *Akodon montensis*, and TK184717 is *Nectomys rattus*. Enlarged view of lesions are shown for TK184521 and TK184717.

Human cases of cutaneous leishmaniasis have been reported in RNBM, including at least two of the park guards (R. Villalba, pers. comm.). Because sigmodontine rodents are common and exhibit a diversity of habits and habitats, we hypothesized that they might include important natural reservoir species for *Leishmania*. Therefore, we included scoring of a tail scar/lesion (**Fig 4**) in our records when the rodent had a tail scar characteristic of cutaneous leishmaniasis [37, 38]. During our review of all 417 rodents in the field, 78 rodents (19%) had visible tail lesions (**Fig 4)**. Seven of the 11 rodent species were affected, including *A*. *montensis* and *O*. *nigripes*.

We examined the association of sex, age, weight, habitats and tail scar with Ab/RNA in logistic regression framework. Female rodents had significantly reduced risk of Ab/RNA compared to male rodents (Odds ratio: 0.36 (0.14–0.92); p = 0.02; **Table 9**). Habitat was a marginal risk factor for infection (p = 0.074). Rodents in High Forest had higher risk of getting infection compared to Bamboo Understory (Odds ratio: 4.83; 95%CI: 1.36–17.14; p = 0.015).

**Table 9:** Univariate association of sex, age, weight, habitat and characteristic tail scar with hantaviral Ab/RNA in captured rodents.

Predictors	No. Mice	Factor Level	No. Negative (%)	No. Positive (%)	Odds Ratio	P Value	LR P Value
Sex	412	Female	181(96.79%)	6(3.21%)	0.36 (0.14–0.92)	0.0327	0.0225
		Male	206(91.56%)	19(8.44%)			
Age	415	Adult	248(92.88%)	19(7.12%)	1.97 (0.57–6.82)	0.2868	0.5138
		Juvenile	64(94.12%)	4(5.88%)	1.6 (0.35–7.43)	0.5457	
		Sub-adult	77(96.25%)	3(3.75%)			
Weight	414		28(3–299)	38(3–60)	1.01 (0.99–1.02)		0.4514
Habitat	395	BF	15(93.75%)	1(6.25%)	2.6 (0.25–26.62)	0.4208	0.0742
		HF	121(88.97%)	15(11.03%)	4.83 (1.36–17.14)	0.0147	
		LiF	73(94.81%)	4(5.19%)	2.14 (0.46–9.82)	0.3291	
		RV	44(95.65%)	2(4.35%)	1.77 (0.29–10.97)	0.5381	
		BU	117(97.5%)	3(2.5%)			
Tail Scar	405	No	306(93.58%)	21(6.42%)	1 (0.37–2.75)	0.997	0.997
		Yes	73(93.59%)	5(6.41%)			

We fitted logistic regression models on all the subsets of five factors for the 381 rodents based on the criteria presented in **S1 Fig**. Akaike information criteria (AIC) selected the best model with sex and habitat as predictors. Sex and habitat had independent prognostic value of infection in the study population (**Table 10**). Adjusting for habitat, female rodent had less risk of getting infected (p = 0.021). Adjusting for sex, habitat was significantly associated with infection (p = 0.043); rodents in High Forest had significantly high risk of being positive compared to Bamboo Understory (Odds ratio: 5.23; 95%CI: 1.46–18.66; p = 0.011).

**Table 10:** Multivariable logistic regression modeling identified sex and habitat as independent prognostic factor of hantaviral Ab/RNA prevalence in rodents.

Predictor	Level	Odds Ratio (95%CI)	P value	LRT P value*
Sex	Female	0.3448 (0.1318–0.9016)	0.0299	0.0209
Habitat	BF	3.2436 (0.3094–34.01)	0.3264	0.043
	HF	5.2265 (1.4637–18.6627)	0.0109	
	LiF	1.7902 (0.3492–9.1773)	0.485	
	RV	1.673 (0.2688–10.4138)	0.5812	

*Likelihood ratio test p value to compare the full model with the model without the factor.

## Discussion

Like most nations in South America, Paraguay is faced with growing environmental challenges. Both of its major biomes (Chaco and IAF) continue to undergo rapid, anthropogenically-driven land cover change. These changes are reflected in land use change (e.g., deforestation and conversion of land from native cover to pasture and crop land). The RNBM provides one of the largest protected fragments of the IAF in Paraguay and presents an increasingly rare opportunity to study rodent communities in their native habitats. We sought to better understand the habitat associations of rodent reservoirs that harbor JUQV and JABV and assess the abundance of JABV and JUQV reservoirs across the RNBM. Characterization of these virus-host relationships within native habitats are essential to building robust biological, computational, or mathematical models of virus maintenance, spillover, and spread within rodent communities. This is the first comprehensive study to determine the natural habitats of two sympatric rodent reservoirs, *A*. *montensis* and *O*. *nigripes*, in the IAF and the prevalence of JABV/JUQV in these rodents by habitat.

In agreement with reports published by us and others [12, 23, 28, 31], this study affirms that *A*. *montensis* and *O*. *nigripes* are the reservoirs for JABV and JUQV, respectively. We did not find evidence of hantavirus infection in any of the other rodent species captured including the closely related *O*. *mattogrossae* or in *H*. *megacephalus*, the second most abundant species. Previously, we have reported spillover of JUQV in *O*. *mattogrossae*. *Oligoryzomys mattogrossae* has been associated with Anajatuba virus in Brazil [31]. We report that *A*. *montensis* and *O*. *nigripes* have different habitat preferences although there is overlap and hence opportunity for spillover with each other and *H*. *megacephalus*.

The enormous diversity and poorly understood distributions of the sigmodontine species in South America present taxonomic challenges in the identification of the species [9, 45]. We applied molecular methods to supplement field identification, which worked well although relatively few vouchered sequences are available for some species for comparison with our sequences. We found the COI marker worked very well in identification of *A*. *montensis* but did not discriminate species very well for *Oligoryzomys* species. Only the CytB gene differentiated *O*. *nigripes* from *O*. *mattogrossae*. The combined morphological and genetic approaches in taxonomy distinguished 11 rodent species in this study. Species evenness was low. Some species (e.g., *Scapteromys aquaticus* or *Cerradomys scotti*) were represented very rarely, and therefore their association (if any) with hantaviruses is still unknown. Communities with low evenness and high abundance of the primary host have been suggested to present favorable conditions for intraspecific contact, leading to increased probability of virus transmission [46]. The biological diversity of mammalian communities can affect reservoir species-pathogen prevalence, including hantaviruses [47–49]. Often, a dilution effect is observed in which as species diversity increases in a community, pathogen or pathogen-related disease prevalence of infection with the pathogen decreases [48]. This could be due to a variety of mechanisms (e.g., reduced host contact, variation in host competence). Although a general pattern indicated that a higher proportion of RBO-HV occurred in habitats with lower species diversity, we did not find clear evidence of a dilution effect in this field study. At RNBM, rodent species diversity varied with forest type, although some habitat types (e.g., Cerrado) were under-represented in the transect coverage and number of captures.

Although not investigated in our field efforts, dilution effects may emerge with seasonal changes in rodent community diversity. A negative relationship between SNV infection prevalence in *P*. *maniculatus* and species diversity was detected using spring and fall sampling in the USA [47]. Our field study occurred during the Paraguayan winter (August), and therefore future investigations could address seasonality in RBO-HV infections, its importance for viral maintenance in the rodent populations, and in potential transmission risk to humans. Similarly, fluctuations in the overall rodent community structure should be considered, because temperature, climate and/or resource availability can affect population dynamics of small mammal species (for example see [50]) and prevalence of RBO-HV. Teixeira et al. (2014) found infection of *A*. *montensis* with JABV throughout the year in southern Brazil with peaks during warmer months (December–March) [51]. In Argentina, peak abundance in rodent communities occurred during winter months (June to September) [46]. Our prior studies of *A*. *montensis* and JABV did not find a correspondence between RBO-HV antibody prevalence and seasonal variation in the RNBM, however, we noted the sex ratio varies seasonally in *A*. *montensi*s [52]; however, seasonality was not included in the design of this study.

*Akodon montensis* predominated in this forest ecosystem assemblage. This species has a large distribution ranging from Argentina, Brazil, Paraguay and Uruguay, and harbors JABV and Apé Aime (a reassortment of JAB and Pergamino hantaviruses) [52, 53]. The second most common species, *Hylaeamys megacephalus*, a larger rodent found at Mbaracayú, presented no evidence of RBO-HV infection; and none to our knowledge has been reported in other studies. In addition to the rodents, three marsupials, *Gracilinanus agilis*, that were captured during our study, were negative by IFA.

As expected, evidence of hantaviruses was more frequently detected in male rodents during our study. A higher seroprevalence in older male rodents has been reported in North and South America (e.g., SNV and *P*. *maniculatus*, *P*. *boylii*; Bayou virus in *Oryzomys palustris*, ANDV and *O*. *longicaudatus*) [3, 44, 54–57]. In our study, age group did not influence the prevalence of hantaviral antibodies, however we found only adults to have hantaviral RNA (10 of the 11 being male) suggesting that active virus transmission may occurs primarily among males. Mills *et al*. (2005) highlighted behavioral factors including fighting or communal behavior, as a regular condition of zoonotic virus prevalence and transmission [58].

We identified three antibody-positive juveniles. This might be due to transfer of maternal antibodies from infected dams, and neonatal pups have been found with passively acquired transferred antibodies via milk, for up to two months [59]. However, Taruishi *et al*. (2008) found no evidence of vertical transmission of Hantaan virus in lab mice [60], and hence, passive transfer of antibodies is the probable route. Calisher *et al*. (2009) discuss various mechanisms for young rodents to become naturally infected by hantaviruses, such as communal nesting with exposure to excreta of an infected adult [61].

We examined the general health status of each rodent and recorded the visual presence of tail scars suggestive of *Leishmania*. Leishmaniasis is endemic in Canindeyú Department where RNBM is located [62, 63]. We did not find lesions*/*scarring to be associated with hantaviral Ab/RNA status. Gross pathology of rodents during necropsy suggests additional diseases and/or endoparasites were prevalent and potentially impact the health of IAF rodent communities; further investigation of these diseases is warranted.

In conclusion, in contrast to findings from studies of RBO-HV populations in Central and North America, our analyses of species diversity did not show an association with the presence of RBO-HV. Comparing across the eight habitats sampled there was no apparent linear correlation between rodent species diversity and hantaviral prevalence. The habitat distribution data suggest that there may be a possibility for spillover; however, in our prior studies and this study, JUQV has not yet been observed in *A*. *montensis* or JABV in *O*. *nigripes*. However, as mentioned in the introduction, a JUQV-like virus, Araucaria hantavirus, has been identified in *A*. *montensis* [34]. By characterizing rodent population structure, demographics and habitats, together with their association with hantaviral prevalence at RNBM in northeastern Paraguay, we have a better understanding of the habitat preferences of two widespread, reservoirs of RBO-HV. Future longitudinal work will focus on how these microhabitats support RBO-HV maintenance, spillover, and interactions that promote transmission within and perhaps between the rodent species communities. This is important, as we have limited information on the natural habitat preferences of the majority of RBO-HV rodent reservoirs and the biotic or abiotic environmental pressures that promote maintenance or increases in the prevalence of RBO-HV infection. As land-use changes are occurring in and around many forested regions of the world, adopting a One Health approach to integrate wildlife, environmental and human health becomes more relevant. Moreover, given the anthropogenic encroachment on forests such as the IAF and RNBM, it is important to understand the ecological relationship between viral hosts, their environment and pathogen prevalence as humans increasingly interact with such ecosystems.

## Supporting information

S1 Fig Flow diagram of specimens used in analyses. (DOCX)

S1 Table Trap line descriptions and trapping plan. (XLSX)

S2 Table Raw field and laboratory data. (XLSX)
