# Supplementary material for: Habitat, species richness and hantaviruses of sigmodontine rodents within the Interior Atlantic Forest, Paraguay
Source: PLoS One. 2018 Aug 1;13(8):e0201307. doi: 10.1371/journal.pone.0201307 (PMC6070238; doi:10.1371/journal.pone.0201307)
Supplement: S1 Fig — (DOCX) [file pone.0201307.s001.docx]

417 mice captured

2 mice had no IFA

381 mice included in multivariable model building

Ce (4), LF (8) and MG (8) had less 10 mice and were excluded in logistic regression model involving habitat.

3 mice missed sex

1 mice missed age

10 missed leish

395 mice are included in univariate logistic regression model with habitat as independent variable

415 mice included in description distribution of mice and prevalence of hantavirus

S1 Fig. Flow chart of mice included in the study
